# Vulnerability and threat: describing gay male victimologies in South Africa by analysing online community reports

**DOI:** 10.3389/fsoc.2025.1539410

**Published:** 2025-02-14

**Authors:** Marchant Van Der Schyff

**Affiliations:** IIE's Varsity College, Sandton, South Africa

**Keywords:** gay men, gender-based violence, framing, online news media, queercide, Grindr

## Abstract

This study examines the phenomenon of violence towards gay men in South Africa, focusing on its portrayal and understanding within online media. Using a qualitative approach, it explores how online reports construct, interpret, and contest narratives surrounding these attacks. The investigation aims to highlight the portrayal of the victimology of gay men and the societal dynamics, including gender-based violence, abduction, victimisation, and queercide, in the South African context. The research analysed online reports, between 2022 and 2024, by a community organisation on cases of violence perpetrated against gay men. The research found that media frames about the circumstances of violence, the role of social media or hookup apps, and demographic details of gay men can provide insights into their victimhood. This contributes to the understanding of the challenges gay men face in navigating their identities and safety in South Africa. The findings show that Gauteng, Cape Town, and Durban remain the most affected by attacks against gay men and provide more insight into the strata of the age of victims, their movement, and the nature of attacks. By identifying how online community media frames these cases, the study offers insights into the emergence of gangsterism and the use of mobile technologies to target these men. It can inform strategies for social change and foster inclusive environments for marginalised communities. The findings have implications for advocacy, policy making, and community empowerment efforts addressing queercide and promoting LGBTIQ+ rights and safety.

## Introduction

The LGBTIQ+ community “who are already stigmatised for other reasons of minority stress” – Terrance Crawford, Director of Crystal City documentary (This Pink Cloud, 2022) is being victimised, including occurrences of violence perpetrated against gay men in new socio-technological environments that require renewed and continuous research (De Barros, 2024). The origin of judgemental views about gay men, causing them to be more vulnerable, is arguable. Contemporary ideas could be influenced by religious or cultural beliefs that same-sex attractions are sinful, or that in many countries around the world, antiquated sodomy laws to gay male relationships, still exist. The social authority required for gay men to live empowered lives with dignity, free from the pervasive threat of violence remains mostly ephemeral or inaccessible (Yates, 2022). Pontifications about what normal sex and sexual orientation are, with heteronormativity as the default, are a challenge to emancipatory conscientisation about the LGBTIQ+ community and thereby continue to legitimise homophobic behaviour, including physical violence against gay men (Poiani and Dixson, 2010). In an article contextualising “victimology,” Oakes-Odger (2025) claims that perpetrators (of gender-based violence) select their victims based on the level of perceived susceptibility this community has and that victims are somewhat complicit in the violence perpetrated against them due to allowing prejudicial views to persist. This article recognises that socio-cultural contexts influencing the violence being committed against gay men exist who, as a result, remain vulnerable because of it (Dresden, 2021). Queercide and other forms of homophobic incited violence are especially prominent on the African continent for example in Uganda, Nigeria, Tanzania, Ghana, Egypt, Cameroon, Senegal, and Kenya, while in South Africa LGBTIQ+ persons continue to be vulnerable to violent attacks according to the Human Rights Watch (2023) trends report. Because of discrimination, many African gay men are fleeing to South Africa as refugees with the hope of being safeguarded against physical aggression (York, 2023). However, the reality is that LGBTIQ+ refugees as well as South African gay men do not feel protected (The Pink Cloud, 2022). A survey by the World Economic Forum on “attitudes towards homosexuality and gender non-conformity in South Africa,” found that even though 51% of South Africans believe that gay people should have the same human rights as their heterosexual peers, 72% feel that same-sex activity is morally wrong. The survey also found that 44% of LGBTIQ+ respondents experienced bullying, verbal and sexual discrimination, as well as physical violence in their everyday lives due to their sexual orientation (Pillay, 2018). As part of the LGBTIQ+ sub-culture, gay men are experiencing abductions, torture, and queercide. Incidences of homophobic violence are unique to this community, and a better understanding of their victimology and information around these incidences will help to address this vulnerability (Herek, 1984; Igual, 2023a).

### South African community media coverage of gender-based violence

One way of addressing violence against the LGBTIQ+ community is by generating supportive media exposure as well as evaluating how the media portrays these cases of brutality (Schifter, 2020). The influence of media is that they can guide how audiences think about an issue, determining how (and which) issues are discussed by framing, representing, and reflecting crimes based on sexuality and discussing which perspectives are omitted (Goffman, 1974; McCombs, 1977; Breen, 2004). For example, online community media could decide to report on a victim’s profile but could also preface it with a focus on the perpetrator(s) and soft measures, such as gun control or the use of dating apps, depending on what motivates their philosophy about newsworthiness (Valcore and Buckler, 2023). Another example is that there are no online media reports found from *Google* searches, from either news- or community- online media, on the recent disappearances of South African gay males including Gerhard Naude, Ntokozo Zuke, or Wayne Johnson even though there has been a spike in these cases (Igual, 2023b).

As an important consideration in the understanding of South African gay male victimologies, using online community reports to investigate *who* the perpetrators of violence are provides the reader with a broader view of the harmful effects of crime. For example, it might be easier to assume that perpetrators of gender-based violence have suffered harm and abuse as children, while an article by Johnson (2023) presents statistical arguments that 74% of surveyed hate crime offenders in a Scottish Prison committed these acts due to witnessing bigotry and hate in their families and communities towards LGBTIQ+ persons. Oaks-Odger (2025) also express the necessity of evaluating the victim by including the perpetrator’s profile. Therefore, this study looks at what we understand about South African gay male victims and their habits as it is uncovered in the frames that the online community media use to report on it. The answer may contribute to a fundamental awareness of how to improve social justice and safety for these men while unmasking malefactors in the process.

### Methodology

This qualitative investigation contributes to the understanding of South African gay male victim profiles as embedded in the online reports on gender-based violence by an online community platform. The research describes reported cases of brutal violence to identify emerging information on the victimology of these men, including their movement, age, location, nature of the attack, and occupation, as well as the role technology played in each case, the recommendations to reduce the vulnerability of these victims, and the profile of the perpetrator(s). The study was done through a desktop research design to collate existing and accessible online reports in which relevant documents were reviewed. Although the measures used to establish the reliability and validity of quantitative studies cannot necessarily be applied to qualitative research, the rigour of this research was established by adhering to Lincoln and Guba (1985) criteria of neutrality, applicability and consistency. For example, I accounted for any personal biases which may influence findings by reading and rereading the selected online community reports to confirm, remove and adjust codes to address any truth biases that may exist. Also, I meticulously kept record of all the reports in the population group, the selection criteria, as well as codes and themes to ensure the soundness of this research.

### Data collection

Using systematic *Google* searches to identify relevant online reports, the initial search employed the terms “violence against gay men” and “South Africa.” The results were varied, making it difficult to discern between valid, useful reports and those less informative. In the secondary search strategy to improve on this variability, additional and clearer prompts were used to yield more focused results. These prompts were developed by considering more specific population characteristics and search parameters. First, the search was limited to LGBTIQ+ specific online publications and news formats to ensure that the sources included subjective epistemic insights and information which may be newsworthy to this community. The definition of “gay men” was clarified to include men who identify as gay—whether transgender, cis-gender, or intersex—but excluded men who have sex with men (MSM) who do not identify as gay. Similarly, “violence” was defined more precisely, including abductions, torture, blackmail, murder or queercide, disappearances, and larceny. This narrower focus helped to refine the search results to more pertinent cases of violence experienced by gay men in South Africa. However, some variance in the results continued thus making comparability problematic.

To further improve the trustworthiness of the data, a third round of searches on reports published between January 2022 and June 2024, 18 months, was performed. Additionally, LGBTIQ+ publications were prioritised to ensure that the data was current, community-centred, and reflective of the lived experiences of gay men. After refining the search criteria, the search yielded reports from a range of LGBTIQ+ publications, including *Gay Pages SA*, *MOFFIES*, *The GALA Queer Archives*, *OUT: LGBT Wellbeing*, *Out Africa*, and *MambaOnline*. However, due to their distinctive purposes and formats—for example, the health-focused *OUT: LGBT Wellbeing* or the lifestyle-oriented *Out Africa* —I chose to focus solely on *MambaOnline*, as it consistently provided news-reporting content relevant to the study. *MambaOnline*’s reports were deemed more suitable for comparison and analysis within the context of violence reporting.

The third round of searches produced 34 relevant reports that met the established population criteria. From this pool, a sample of 11 reports was selected using a probabilistic simple random sampling method, whereby every third report was chosen to reduce the number of units for analysis. The population criteria, including the date range and nature of the article, were applied to the complete news article archive of the LGBTIQ+ online community website *MambaOnline*, which yielded relevant reports. Although the prevalence of sampling errors and selection bias can be reduced by increasing the sample size to get closer to the actual population, this qualitative study intended to describe the victimologies of gay men in South Africa and not the replicability or generalisability of the results. This method ensured the selection was unbiased and allowed for a representative and manageable sample for analysis.

To improve the reliability of the selected reports, each case was fact-checked on other, reputable online news sources. For example, in Article 1 *Three Cape Town Grindr gang suspects appear in court* (Igual, 2024a) the case was also reported on by IOL Reporter, Tshwete (2024) in his article *Robbers trap “lonely” gay Grindr user*, as well as Cape Town Etc’s Daries (2024) in his article *Three arrested in Woodstock for Grindr robberies*. In this way, the sources were verified, however, some potential sampling biases could have influenced the representativeness of the 11 reports. Through time and care, I tried to understand the population thereby reducing sampling bias as much as possible.

### Data analysis

I performed a thematic analysis to link themes to the data from the online reports (Braun and Clarke, 2013). For example, an extract from the online report: “In early April, an LGBTIQ+ individual from Inanda was beaten, strangled, and robbed…” (Igual, 2024c) is directly linked to the *nature of the attack* and *location* of the *victimology* theme. In this type of analysis, it is also typical to frame the themes through theoretical epistemologies, therefore, the study included relevant principles from Queer Theory as conceptualised by Michel Foucault, Framing Theory, and Life Course Theory. These theories situate the results to answer the research question. The utility of the three theoretical frameworks changes the way the data around victimologies are analysed, for example, Queer Theory emphases societal norms, systematic oppression, and how queer identities intersect with violence. Life Course Theory critiques the developmental transitions and long-term effects of gay male victims of violence, while Framing Theory focuses on how gender-based violence perpetrated against these men are presented and potentially shapes public and institutional responses. By immersing myself in reading and rereading the online community reports, I identified possible themes by grouping codes, highlighting, and assigning annotations to them. From these data-driven codes, themes emerged to analyse the codes (Braun and Clarke, 2013) in a process of praxis. The themes revealed that there is more data available than merely describing the *victimology* (theme 1) necessary to understand the victim profile and vulnerability to gender-based violence of South African gay men. These emergent themes include *the role technology played in incidences of violence* (theme 2), *recommendations for reducing vulnerability* (theme 3), and *perpetrators’ profile* (theme 4).

Table 1 provides an analysis of *victimology* (theme 1), including the *age of victims* (theme 1.1), *location where victim was attacked* (theme 1.2), *nature of attack against victim* (theme 1.3), the *reason(s) for the vulnerability of the victim* (theme 1.4), and *movement of victims* (theme 1.5) to provide more insight into the profile of South African gay men who have been victims of gender-based violence. One trending theme is the frequent targeting of younger victims in their twenties (Articles 4, 6, 8, 9, and 11), though victims as old as 41 and 43 also appear (Articles 2, 5, and 10). Many attacks, as reported by the selected community online reports, occur in urban settings, especially in Johannesburg (Articles 2, 4, 5, 6, 7, 8, 9, and 11) and Cape Town (Articles 1, 2, and 6), highlighting geographic patterns of vulnerability. Victims are often lured to either the perpetrator’s indicated residence or an out-of-home area under the appearance of meeting up (Articles 1, 2, 3, 7, 8, and 11). The nature of the attacks ranges from strangulation and beatings to kidnapping and blackmail (all Articles), with some victims being filmed naked or held for ransom (Articles 1, 8, and 9). In Articles 1, 2, and 3 the reasons for vulnerability are described as the failure of law enforcement response, as police are described as dismissive or slow to act, while none of the remaining Articles mention any specific reason why South African gay men are more vulnerable to gender-based violence.

**Table 1:** Detailed information from online community reports of South African gay men who were victims of gender-based violence.

	Theme 1: victimology				
	Sub-theme 1.1: age of victim	Sub-theme 1.2: location where the victim was attacked	Sub-theme 1.3: nature of attack against the victim	Sub-theme 1.4: reason(s) for vulnerability of the victim	Sub-theme 1.5: movement of victim
Article 1 (Igual, 2024a)	Not reported	Cape Town	Strangled, tied up, stripped naked and filmed; threatened; robbed of R3 000; victim’s family terrorised	Police showing little interest in investigating these cases	Lured into meeting for a coffee date at the perpetrator’s residence
Article 2 (Igual, 2024b)	43	Cape Town; Gauteng (Johannesburg)	Two victim reports: Victim 1 - tied up with shoelaces and socks, stripped naked and filmed; Attackers threatened to kill, dismember and sell victim’s body parts; raped. Victim 2 - strangled to death	Law enforcement was not helpful - dismissed and kept redirecting the victim	Victim 1 - lured to the perpetrator’s location; Victim 2 - lured to a guest house
Article 3 (Igual, 2024c)	Not reported	KwaZulu-Natal (Durban)	Beaten, strangled, and robbed	Police initially responded unprofessionally	Not reported
Article 4 (Igual, 2024d)	28	Johannesburg	beaten, robbed, and stabbed in the stomach; stripped naked and filmed; blackmail	Not reported	Not reported
Article 5 (De Barros, 2024)	43	Victim 1 - Durban; Victim 2 – Gauteng (Johannesburg)	Beaten, strangled, and robbed	Not reported	Went to the attacker(s)’ residence (movement)
Article 6 (Mamba Writer, 2024)	Victim 1–21; Victim 2–18	Victim 1 - Delft, Cape Town; Victim 2 – Johannesburg	Victim 1 - Stabbed in the neck and died; Victim 2 - kidnapped, beaten, and ransom demanded	Not reported	Not reported
Article 7 (Igual, 2024e)	18	Johannesburg	Held captive, beaten, ransom of R30 000 demanded from the family	Not reported	Lured the victim to their location
Article 8 (Igual, 2023a)	22	Johannesburg	Kidnapped and held captive for 5 days; ransom of R50 000; terrified friends and family by sending them videos of victim being assaulted	Not reported	Lured victim to attacker’s location
Article 9 (Igual, 2023b)	22	Victim 1 - South of Johannesburg; Victim 2 - Johannesburg	Victim 1 - Held hostage for 5 days; ransom of R50 000 demanded from followers of victim’s social media accounts; terrorising friends and family by posting threats; stripped the victim naked, bound and filmed them; Victim 2 - kidnapped, extorted, beaten and attempted murder	Not reported	Not reported
Article 10 (Igual, 2023c)	41	Uitenhage, Eastern Cape	strangled, beaten, stabbed to death; the body mutilated and set on fire	Not reported	Not reported
Article 11 (Igual, 2023d)	26	Parktown, Johannesburg	Victim was hijacked, kidnapped, and robbed of his personal belongings including bank cards	Not reported	Victim was lured to an area

For the sections where there was “not recorded” data, the themes occurred significantly enough in the sample to be included (thus, three or more of the selected community reports), however, might not have appeared in some of the units of analysis. It can be noted that the most significant absence of data among the selected reports occurred in Theme 4, which highlights the *reasons for victim vulnerability*, where only three reports (27%) included relevant information. This was followed by Theme 5 regarding the *movement* of victims (56%), and Theme 1 which identifies the *age* of the victims (82%). In describing the profile of the victims, it can therefore be determined that a victim’s age is more likely to be reported than the reason they were vulnerable in this context.

### Ethical considerations

Gender-based violence against vulnerable groups, such as gay men, is complex and I had to treat the subject and incidences with considerations of care and understanding. According to Sikweyiya and Jewkes (2011), when researching sensitive topics there is a risk of under-reporting the extent of violence experienced or perpetrated, especially in Southern Africa. Therefore, I expanded on the initial *victimology* theme to be more sagacious about the breadth and depth of the issue.

Conducting Internet-based desktop research, Cilliers and Viljoen (2021) developed a framework for Ethics Committees to use in their decision-making and explain the importance of having a representative study population to address challenges about its ethical use. I used this framework to identify the population of online community reports used to collect data and gain insights into the victim profile of South African gay men. This included iterative processes of selecting and eliminating potential units of analysis which diluted the trustworthiness of the data. The study also received ethical clearance from the Independent Institute of Education’s Ethics Committee in 2024.

## Results

In this Internet-based desktop study, 11 online reports of incidences of violence against gay men from a community focussed organisation were analysed by applying five broad themes. The purpose of the study is to gain better insight into the victims and context of these incidences, which are currently lacking in existing literature, to improve the knowledge of issues affecting the LGBTIQ+ community, especially gay men, and promote social justice.

For the *victimology* theme, Articles 1, 2, and 6 reported that incidences of violence against gay men occurred in Cape Town. Articles 2 and 6 also reported on separate incidences which had occurred in Gauteng along with articles 4, 5, 7, 8 and 9. From the 11 online reports, two violent attacks were reported to have occurred in KwaZulu-Natal (Articles 3 and 5), while article 10 described an incident in the Eastern Cape. Only Article 11 omitted the province where the reported violence occurred. The *ages* of the victims varied between 18 and 28 as identified in Articles 4, 6, 7, 8 and 9, and 41–43 in Articles 2, 5, and 10, while Articles 1, 3, and 11 did not disclose the victims’ ages. The age median of 30 years old (29.6 when calculated) is not as noteworthy as the age strata 22–28, and 41–43 (with one victim being 18). The reason for this can be found in the discussion section of the article. In three of the online reports (Articles 1, 2 and 3) the *reason for the vulnerability* of the gay men was attributed to “the police showing little interest in investigating the reported attack,” while being “dismissive and unprofessional.” The *nature of the attacks* perpetrated against the victims included torture (Articles 1 “threatened,” 2 “tied up with shoelaces and socks”; “threatened to kill, dismember, and sell body parts”; “strangled,” 3 “beaten and strangled,” 4 “beaten and stabbed in the stomach,” 5, 6 and 7 “beaten,” 8 “being assaulted,” 9 “bound and beaten so severely that he almost died,” and 10 “beaten and strangled”), robbery (Articles 1 “stole R3 000″, 3, 4, 5, 6, 7, 8, and 9), being stripped naked and filmed (Articles 1, 2, 4, 8 and 9), abduction (Articles 6, 7, 8 “held for 5 days,” and 9), terrorising the victim’s friends and family (Articles 1, 6 “demanded ransom from friends and family,” 7 “demanded R30 000 ransom from family,” and 8 “demanded R50 000 from family”; “posting threats to the friends and family of victims on their social media”), murdered (Articles 2 “strangled to death,” 6 “was stabbed in the neck and died,” and 10 “stabbed him to death, mutilated the body and set it on fire”), and rape/ sexual assault (Article 2). In five of the online reports, the *movement of the victim* was described as: “lured into meeting the perpetrator at their residence” (Articles 1), or “lured to a guesthouse” (Articles 2, 5, 7, and 8). Finally, Article 4 described the *length of time* each victim engaged with the perpetrator(s) before a physical meeting where the attack occurred by stating: “some victims engaged with their attackers via video- and phone calls for weeks.”

The online reports also described how *technology* was used in the attacks against gay men. Dating apps (“Grindr” mentioned in Articles 1, 3, 7, 8, 9, and 11; “Surge” and “Facebook” in Article 1) and the victim’s phone (“the attackers used the victim’s phone to blackmail them and, by extension, terrorise their friends and family” in Article 1; “victim’s phone was used to access their bank account, change their social media and financial account passwords, and creating fear by accessing and communicating with the victim’s friends and family in Article 5).

Most of the online reports provided *recommendations for decreasing the vulnerability of gay men* by advising them to “read and consider online dating and hookup safety tips” (Articles 1, 3, 5, and 9), “to remain vigilant (Article 3), and “to remain outraged about these attacks against gay men so that they are not normalised” (Article 7). None of the online reports described *changes in the gay sub-culture* that might contribute to their vulnerability to attacks. However, they do confirm that there is a “surge of violent attacks and deadly abductions and robberies due to social stigma” (Article 1) and therefore the victim being “less likely to reports the attack” (Articles 1, 3, 9, 10, and 11) as well as “secondary victimisation by authorities” (Article 1). The online reports also state that gay men are “increasingly using dating apps” (Articles 2, and 3) providing more opportunities for criminals to target them using these platforms.

Many of the articles applied the economy of scale, using the same reporting information from different cases, for example, Article 1, reporting on a kidnapping case in Cape Town (Igual, 2024a) and Article 2, reporting on a syndicate targeting gay men in Gauteng and Durban (Igual, 2024a), such as the section on *victimology: what makes gay men more vulnerable?* Here, the following section was used: “more vulnerable due to social stigma, and because they are less likely to report the attacks out of fear of being outed, shamed, or facing secondary victimisation from the authorities.”

## Discussion and recommendations

Using the principles of Queer Theory, grounded in Michel Foucault’s ideas on power, sexuality, and the regulation of marginalised identity, the study’s findings clarify how gay men are predisposed to gender-based violence not only because of prejudice but also due to structural forces (Sawicki 2010, in O’Leary and Falzon, 2010). Two key questions about gaining insights into the victimology of South African gay men include how heteronormative societal norms and structures perpetuate violence against gay men, and how the unique forms of victimisation this group face are due to their sexual orientation. Foucault’s ideas on power and identity critique how societal norms and structures construct and regulate sexualities to negatively impact those who deviate from heteronormativity. In this study, Foucault’s concept of biopolitics – where the government exercises control over individuals – can be observed in how the South African Police Service (SAPS), which is an agent of the government, does not protect gay men through a lack of urgency in dealing with reports of gender-based violence. Their unprofessional and indifferent behaviour fosters a fear of reporting due to secondary victimisation (as shown in Articles 1, 2, and 3) and limits gay men’s ability to seek justice. This is indicative of broader societal mechanisms that marginalise the LGBTIQ+ community. This stigma allows criminal entities to exploit this susceptibility. Practical recommendations which could address societal reform, according to an Australian Government Gazette article by Koonin (2024), is for members of the LGBTIQ+ community to have a platform to express the trauma and hurt which affects them and how this influences the distrust they have of government services. They continue to describe that statements of apology, which recognise the suffering and trauma experienced by gay male victims, survivors, friends and family, as well as the broader LGBTIQ+ community will be a critical juncture in a long-term journey towards justice, equality, and healing. There also needs to be a taskforce working alongside LGBTIQ+ consultive committees and SAPS to oversee the implementation of police-related recommendations in a timely manner (Koonin, 2024). Although these recommendations originated from Australia, South Africa has benefitted from similar tactics in the 1990s during the post-Apartheid Truth and Reconciliation Commission.

Queer Theory also applies to the use of dating apps, such as *Grindr* and *Surge* (in Articles 1, 3, 7, 8, 9, and 11), and technology in these exploitations as gay men, in particular, are open to new digital spaces of risk. Criminals are using their victims’ phones to blackmail and terrorise them. This further exemplifies the way technology extends gender-based violence and sociocultural and institutional control over gay men’s lives. This highlights Foucault’s ideas about how power does not only repress but also produces the conditions of visibility and silence, allowing conditions for gender-based violence to persist (Sawicki, 2010, in O’Leary and Falzon, 2010). Foucault’s principles are relevant to investigating how societal norms around sexuality and identity contribute to the victimisation of gay men.

Other assumptions that help us understand how incidences of gender-based violence against South African gay men are framed in the media, which biases or stereotypes are present in these frames, and how they describe the experiences and treatment of gay male victims, is the Framing theory developed by Ervin Goffman in 1974 (Scheufele, 1999). Certain aspects of the online community reports that are emphasised can influence the reader’s interpretation of these incidents. The online community reports from *MambaOnline* frame violence against gay men in a way that focuses on their victim profile and vulnerability, the role of technology in facilitating attacks, and the failures of the police service. By using these lenses, the reports depict gay men as targets of violence due to systemic neglect, and their use of specific digital platforms, such as *Grindr* and *Surge*. The online reports refer to the police being “dismissive and unprofessional” and showing “little interest” (Articles 1, 2, and 3) further contributing to the vulnerability of South African gay men. This framework highlights the SAPS as a part of the issue of injustice experienced by the victims. Similarly, the references to dating apps as a platform used by criminals to lure victims and thereby focus attention on how technology contributes to the risks faced by gay men (Articles 1, 5, 7, and 8). The framework creates an intersection of gay men’s online behaviour and their physical vulnerability (Fairhurst and Sarr, 1996). The online reports also contribute to the perception that gay men are less likely to report incidences of gender-based violence due to the fear of being shamed, outed, or subject to secondary victimisation (Articles 1, 9, and 10). This social stigma framework situates the violence within a larger societal context of homophobia, where the reader is encouraged to understand the attacks as a part of a broader pattern of socio-cultural marginalisation instead of isolated incidents.

The limited attention to potential risk factors within the gay sub-culture suggests a framework that situates the blame for South African gay men’s vulnerability externally, on social structures and threats, rather than on sub-culture dynamics. These socio-cultural and technological frameworks starkly contrast with those used in recent films and television series. According to Lodge (2024) these audio-visual media provide broader exposure to the rising chemsex phenomenon in the gay male community and how this is a large contributing factor to their vulnerability describing the source of gender-based violence as being internal. The online report frameworks therefore suggest a strategic exploitation of trust in the police, or socio-cultural conventions by perpetrators. In referring to these frames it provides a critical perspective which, according to Lindgren et al. (2024), addresses societal resistance to stories about violence against gay men by understanding how this issue is presented through the media. Framing Theory postulates that frames are used most effectively in persuasive messages – to create awareness and support interventions – when they consider readers’ preexisting opinions and negativity bias in truth perceptions (Lindgren et al., 2024). The importance of online community reports in people’s lives is also supported by Metula (2023) who writes that the information helps to develop their communities, such as members of the LGBTIQ+ group, by highlighting what is of importance to them. Based on the discussion, some recommendations for improving the safety of South African gay men could be sensitivity training of SAPS Officers through building partnerships with relevant social organisations. From Articles 7 and 8, the community-specific social organisations include *Access Chapter 2*, *OUT LGBT Wellbeing*, and *Parents, Families and Friends of South African Queers (PFFSAQ)* (Igual, 2023d; Igual, 2024c). Also, from Articles 1, 3, 5, 7, and 9, there are some practical tips for South African gay men to follow to lower their risk of gender-based violence. These include:

- To read *MambaOnline*’s “Online dating and Hook-up safety tips” found on their website (De Barros, 2024);
- To read *Grindr*’s “Safety and Privacy guidelines” which can be found when opening the dating app’s landing page as well as *MambaOnline*’s website (De Barros, 2024);
- To stay vigilant when meeting others through dating apps by asking discerning questions and by sharing the details of meetings with a trusted friend or family member (Igual, 2024c), and by meeting the person one is engaging with multiple times in public, before going to their residence or inviting them to yours (De Barros, 2024); and
- To reach out for clarity and support to uphold LGBTIQ+ Rights, or to lodge a complaint about unfair discrimination by e-mailing *OUT* at report@out.org.za (Igual, 2024c).

From the online community reports, other key questions to understanding the South African gay male victim profile, include how victimisation impacts these men at different stages of their lives, and how their life course transitions, such as coming out, ageing, partnerships, and intersects with experiences of victimisation. Life Course Theory, which focuses on how individual behaviours and experiences are formed over time by various social contexts, can offer insights into the results of this study. This theory posits that victim profiles are not static, but rather influenced by turning points across a person’s life. For example, the study of the victims’ age in the study ranges from 18 to 43 years old, with most of them being in their 20’s. This life stage is typically associated with identity formation, increased social exploration, and increased use of dating apps (Laub et al., 2017). For younger gay men, their engagement with dating apps (such as *Grindr* and *Surge*) and socio-cultural settings are seen as a turning point in life transitions that increase their risk profile, such as being lured into dangerous situations by criminals (Articles 1, 5, 7, and 8). Life Course Theory also suggests that an accumulation of traumatic experiences can lead to greater victimisation over time. For this study, repeated experiences of police neglect and the use of technology to exploit gay men could make gay men more susceptible to violence. These incidents of violence, if unreported or dismissed by authorities, can cumulatively reinforce a pattern of risk over the life course of gay men, as highlighted in Articles 1, 2, and 3 (Laub et al., 2017). In this way, Life Course Theory helps to describe how the interaction between age, life transitions, and systemic factors such as social stigma and police indifference contribute to the victimisation of gay men, with life events – like using dating apps or seeking new relationships – serving as moments of increased vulnerability. According to Life Course Theory, the weaker social bonds gay men establish may explain their antisocial behaviour across adolescence, thus 10–24 (WHO, 2024) which somewhat coincides with the reference to *age* of 18–28 (Articles 4, 6, 7, 8, and11), as well as adulthood, which coincide with the victim profile of 41–43 (Articles 2, 5, and 10).

By intersecting Framing Theory, Queer Theory, and Life Course Theory, researchers can develop a multidimensional understanding of the victimisation of gay men, addressing the complex interplay of societal narratives, identity framing in the media, and temporal dynamics in shaping their experiences. The findings on the nature of the attacks (Theme 1.2) and the role of technology (Theme 2) therefore aligns with principles around how incidences of violence are reported and why South African gay men are more vulnerable to attacks. These principles and findings are further supported by Colliver (2023) who states that the perception of risk associated with gay men using, for example, the Grindr dating app might be reduced through interventionist strategies, but that it does not reduce the actual risk of abductions, threats, beatings, and strangulation a victim experiences when engaging in real-world meetings.

From the findings, some recommendations to inform rapid intervention efforts for short-term and long-term tactics include the need for training for Educators, Law Enforcement Officials, and relevant civil organisations, who deal with gay men during their formative life transition periods, on empathy, vulnerability, and care for the LGBTIQ+ community’s bespoke challenges (Igual, 2024c; Laub et al., 2017). On the website fundsforNGOs (2024) these institutions can foster an inclusive environment by implementing curricula and training that reflect the diversity of sexual orientations and gender identities. This can include incorporating LGBTQIA+ history into life science classes or offering literature that features LGBTQIA+ characters and themes. Law enforcement also requires training on professional response to victim reports (Igual, 2024b) and the South African Court must issue additional punishment due to aggravating factors in the crime which impact the broader LGBTIQ+ community (Igual, 2023c). Finally, sensitivity training is required for SAPS Officers with an emphasis on deferring to standard operating procedures when receiving a report of violence against gay men (De Barros, 2024). Practically, when advocating for new legislation, it is equally important to ensure that existing laws are being enforced effectively. The collaboration between governmental bodies and advocacy groups is necessary to ensure this. Furthermore, public awareness campaigns can educate LGBTIQ+ communities about their rights under these laws and can empower gay men to stand up against discrimination and violence when it occurs (fundsforNGOs, 2024). Johnson (2023) also recommends systemic family psychotherapy and psychological treatment for both perpetrators and victims of gender-based violence in a way we prevent crime and respond to it.

However, some advances to the protection of gay male vulnerability have been made. *Grindr for Equality* executives met with survivors of the ongoing epidemic of Grindr Gang attacks in South Africa, listening to their traumatic experiences and discussing potential solutions. Survivors shared harrowing stories of being lured into fake dates, being abducted, and then subjected to violence, robbery, and threats. The criminals’ callousness and brazenness, along with police incompetence and indifference, were highlighted at these meetings to stimulate strategies to improve the safety and security of the LGBTIQ+ community. Ideas were exchanged on enhancing user protection, including “profile authentication and reporting mechanisms” and confirmations that “South Africa has been plagued by an ongoing epidemic of violent and sometimes deadly abductions and robberies targeting members of the LGBTQ+ community on dating apps and sites for several years” (Writer, 2024).

Violence against the LGBTIQ+ community requires a more sagacious consideration of the term “hate crime,” especially in law enforcement, and in particular, sentencing of perpetrators. Currently, the courts do not discern between crime and hate crime (York, 2023) and in those instances where homophobia is acknowledged in the Court’s judgement, only the perpetrator(s)’ explicit hate or bias is cited (Little, 2024). However, a person does not have to be proven homophobic to commit a hate crime. Suppose a perpetrator committed a crime against a gay man motivated by their vulnerability. In that case, the focus of the action shifts from an internal locus to that of an external socio-cultural awareness of queer realities. It can therefore also be deemed a crime motivated by hate. Although South Africa has a robust Constitution protecting the safety of gay men, there is a failure in its application. The idea is there, but the execution is too often dismal (Engelbrecht, 2024). The study thereby provides an understanding of the unique vulnerabilities of South African gay men as well as practical recourse to reduce incidences of gender-based violence and improve their safety. From the desktop study, it is also clear that there remains a caveat in understanding the internal sub-cultural nuanced behaviour that predisposes this group to violence, as well as a lack of understanding of the extent of these vulnerabilities. For example, in Theme 2 *the role technology played in incidences of violence*, it was evidenced that gay men use hookup apps such as Grindr to satiate their need for sex, companionship and community, however, there is a lack of information on the breadth of the impact of this technology, or why these men would risk violence and abuse by using them. Therefore, further studies to expand on this remain of principal importance.

## Conclusion

The collected data discloses a range of themes that provide more insights into the predisposition of gay men to attacks by criminal elements targeting them because of their structural and socio-cultural vulnerability. This includes the victimology theme, which refers to the age range of victims in their early adulthood and middle-aged life cycles, and metropolitan areas—Durban, Cape Town, and Gauteng—where most of these attacks are reported, and reported on. That the reported attacks occurred in more urban regions at the expense of media focus on cases of gender-based violence in rural settings, might create the appearance that gay men are more vulnerable in urban-centred areas. Here, the absence of evidence is not evidence of absence and requires further investigation to sufficiently service the diversity of contexts across regions or socio-economic backgrounds in South Africa for all gay men. Due to structural failings and broader societal stigmas, gay men are experiencing vulnerability and exploitation. The *MambaOnline* reports emphasise police inaction and the risks of using online dating platforms as a result of weaker social bonds and the fear of secondary victimisation involving violence against gay men. Some recommendations therefore include sensitivity training of relevant authorities which consider the unique challenges faced by gay men. Partnerships with groups like *Access Chapter 2* can foster empathy. Courts should impose harsher sentences for hate crimes, highlighting the need for a nuanced legal approach to anti-LGBTIQ+ violence.

## Data Availability

The original contributions presented in the study are included in the article/supplementary material, further inquiries can be directed to the corresponding author.
